# Supplementary material for: Five new pseudocryptic land planarian species of Cratera (Platyhelminthes: Tricladida) unveiled through integrative taxonomy
Source: PeerJ. 2020 Sep 4;8:e9726. doi: 10.7717/peerj.9726 (PMC7491415; doi:10.7717/peerj.9726)
Supplement: Supplemental Information 1 [file peerj-08-9726-s001.pdf]

Table S1. Primers used in this study.

Genomic region	Primer name	Sequence	Source
**TNUC813**	TNUCF6	CTTTGGGATACTGCTGG	Present study
	TNUCR6	GCAAAACAAAACCAATCC	Present study
**COI**	BarS	GTTATGCCTGTAATGATTG	Álvarez-Presas *et al*. (2011)
	COIR	CCWGTYARMCCHCCWAYAGTAAA	Lázaro *et al*. (2009)
	COIF	CCNGGDTTTGGDATDRTWTCWCA	Lázaro *et al*. (2009)
**18S rDNA type II**	18S1F	TACCTGGTTGATCCTGCCAGTAG	Giribet *et al.* (1996)
	18S7R	GCATCACAGACCTGTTATTGC	Giribet *et al.* (1996)
	18S4F	CCAGCAGCCGCGCTAATTC	Giribet *et al.* (1996)
	18S9R	GATCCTTCCGCAGGTTCACCTAC	Giribet *et al.* (1996)
**28S rDNA**	28S1F	TATCAGTAAGCGGAGGAAAAG	Álvarez-Presas *et al.* (2008)
	28S6R	GGAACCCCTTCTCCACTTCAGT	Álvarez-Presas *et al.* (2008)
**EF-1a**	EFPlatF	GATTGCYCCWGGYCATCG	Carbayo *et al.* 2013
	EFPlatR	GCRATWGAYTCGTGRTGC	Carbayo *et al.* 2013
**Nd4toCox1**	Kete	CATGGTTTTTGTTCTTC	Solà et al. 2015
	Peten	CCAAAACCACCAATC	Solà et al. 2015

References:

Álvarez-Presas M, Baguñà J, Riutort M. 2008. Molecular phylogeny of land and freshwater planarians (Tricladida, Platyhelminthes): from freshwater to land and back. *Molecular Phylogenetics and Evolution* 47: 555–568. https://doi.org/10.1016/j.ympev.2008.01.032

Álvarez-Presas M, Carbayo F, Rozas J, Riutort M. 2011. Land planarians (Platyhelminthes) as a model organism for fine-scale phylogeographic studies: understanding patterns of biodiversity in the Brazilian Atlantic Forest hotspot. *Journal of Evolutionary Biology* 24: 887–896. https://doi.org/10.1111/j.1420-9101.2010.02220.x

Carbayo F, Álvarez-Presas M, Olivares CT, Marques FPL, Froehlich EM, Riutort M. 2013. Molecular phylogeny of Geoplaninae (Platyhelminthes) challenges current classification: proposal of taxonomic actions. *Zoologica Scripta* 42: 508–528. https://doi.org/10.1111/zsc.12019

Giribet, G, Carranza, S, Baguñà, J, Riutort, M, Ribera, C. 1996. First molecular evidence for the existence of a Tardigrada + Arthropoda clade. Molecular Biology and Evolution, 13(1), 76–84. https://doi.org/10.1093/oxfordjournals.molbev.a025573

Lázaro EM, Sluys R, Pala M, Stocchino GA, Baguñà J, Riutort M. 2009. Molecular barcoding and phylogeography of sexual and asexual freshwater planarians of the genus *Dugesia* in the Western Mediterranean (Platyhelminthes, Tricladida, Dugesiidae). *Molecular Phylogenetics and Evolution* 52: 835–845. https://doi.org/10.1016/j.ympev.2009.04.022

Solà, E., Álvarez-Presas, M, Frias, C, Littlewood, T, Rozas, J, Riutort, M. 2015. Evolutionary analysis of mitogenomes from parasitic and free-living flatworms. PLoS ONE, 10(3), e0120081. https://doi.org/10.1371/journal.pone.0120081
