# Supplementary figures and images for: Five new pseudocryptic land planarian species of Cratera (Platyhelminthes: Tricladida) unveiled through integrative taxonomy
Source: PeerJ. 2020 Sep 4;8:e9726. doi: 10.7717/peerj.9726 (PMC7491415; doi:10.7717/peerj.9726)

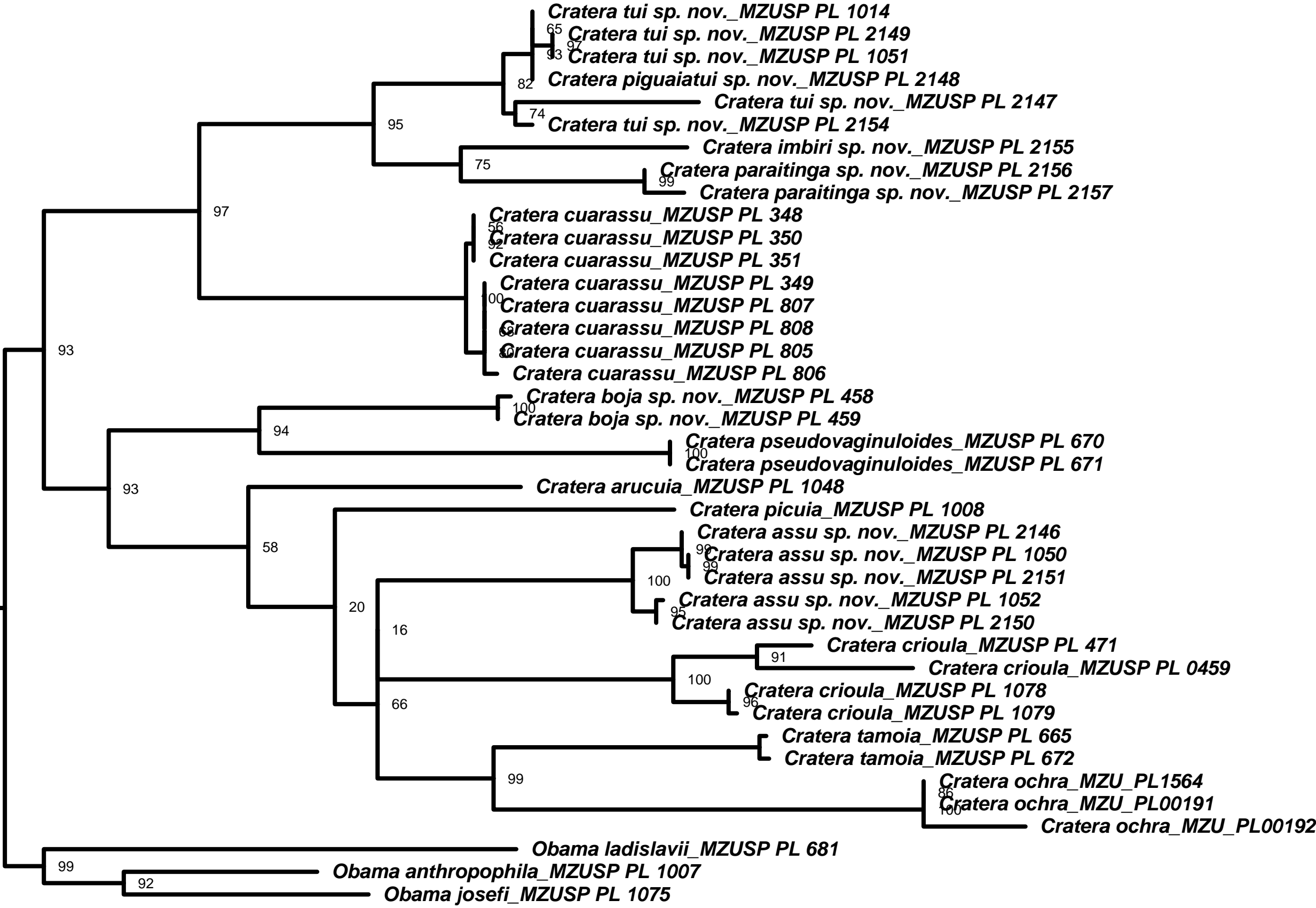

0.02

Supplement: Supplemental Information 2 — Values at the nodes correspond to the bootstrap supports of the analysis. Scale bar represents substitutions per site. [file peerj-08-9726-s002.pdf]
